# Vascular syk-ness: A new role for an old immunological favorite

**DOI:** 10.1016/j.jbc.2024.107517

**Published:** 2024-06-28

**Authors:** Robert Fischer

**Affiliations:** Laboratory of Cell and Tissue Morphodynamics, Cell and Developmental Biology Center, National Heart, Lung and Blood Institute, Bethesda, Maryland, USA

**Keywords:** Syk, vascular, endothelia, ARDS, cadherin, inflammation, fostamatinib, barrier function

## Abstract

Acute respiratory distress syndrome (ARDS) is a deadly clinical presentation in sepsis, COVID, and other lung disorders where vascular fluid leakage is a severe problem. Recent findings by Shadab *et al.* in the JBC show that a well-known player in immune function, Syk, also regulates vascular leakage in response to sepsis. An existing FDA-approved inhibitor of Syk, fostamatinib, prevents the vascular leakage and improves survival in a mouse sepsis model, providing promise for ARDS treatment in the clinic.

Healthy vasculature allows nutrients and fluid to balance with the surrounding tissue and permits immune cell surveillance. For this balance to occur, endothelial cells and leukocytes communicate with each other to prevent leakage of serum macromolecules yet allow leukocytes to pass across the endothelial barrier (extravasation) (1). When these functions are dysregulated, there is increased vascular permeability and aggressive inflammatory responses that lead to disease, including chronic wounds and infections to autoimmune diseases and tumor progression (2). Understanding the communication between endothelial and immune cells in healthy and diseased tissues has been the focus of considerable research efforts. Despite these efforts, some vascular inflammatory disorders continue to elude comprehensive treatment.

One such disorder is acute respiratory distress syndrome (ARDS), where pulmonary edema occurs due to direct physical damage to the lung tissue, aspiration of gastric contents, pneumonia (viral or bacterial), or non-pulmonary sepsis (3). ARDS has been a prominent contributor to COVID-19 mortality. The central issue in ARDS is the loss of endothelial barrier function, exacerbated by inflammatory responses of the immune system. Reduced endothelial barrier function not only results in the frank presentation of edema (fluid leakage) but also decreased oxygen and nutrient delivery, thus increasing tissue damage and further immune recruitment (2). The severity of these events in the lungs is underscored by the ∼40% mortality rate in patients with ARDS (3). Given the limited therapeutic successes in ARDS patients, investigation into new treatments is critical.

While endothelial barrier function is regulated by several mechanical and cellular signaling pathways, a central player in paracellular permeability is VE-cadherin, which mediates homotypic cell-cell adhesion (1). Phosphorylation of VE-cadherin at multiple sites can modulate its association with adapter molecules, which integrate cell-cell adhesions into the actin cytoskeleton and can regulate cleavage or internalization of VE-cadherin (4). Some phosphorylation sites such as Y731 are constitutively phosphorylated, and when dephosphorylated promote leukocyte diapedesis by transient destabilization of the intercellular junctions (1). Conversely, other sites such as Y658 and Y685 are phosphorylated in response to inflammation and cytokine signals by Src and other protein kinases (4). While Src-mediated phosphorylation can induce VE-cadherin internalization, it is insufficient as the sole cause of barrier loss, indicating that there are further complexities in the regulation (5).

The non-receptor tyrosine kinase Syk is a key regulator of the cross-talk between immune cells and vasculature in ARDS as mentioned above. Syk participates in a range of immune cell responses such as B-cell activation, macrophage phagocytosis and cytokine secretion, neutrophil extracellular trap formation, platelet aggregation, and mast cell activation (6). Syk performs this panoply of functions by binding to immunoreceptor tyrosine-based activation motifs common to several cell surface receptors, including B-cell receptors, C-type lectin receptors, and immunoglobulin Fc receptors (6, 7), leading to activation of downstream signaling (Fig. 1). Given this range of immune cellular regulation, it is not surprising that Syk activity plays a role in numerous immune-related pathologies, such as thrombocytopenia, rheumatoid arthritis, COVID-19, ischemia–reperfusion injury, and some types of cancer, in particular leukemias (6, 7). Thus, Syk was an early therapeutic target, yielding several clinical trial worthy candidates, the most promising of which was fostamatinib/R788 that was developed over a decade ago (7). The use of fostamatinib has had some success in the treatement of COVID-19, in part due to its ability to inhibit the Sars-COV-2 induced macrophage hyperinflammation and endothelial disruption (6).

**Figure 1:**
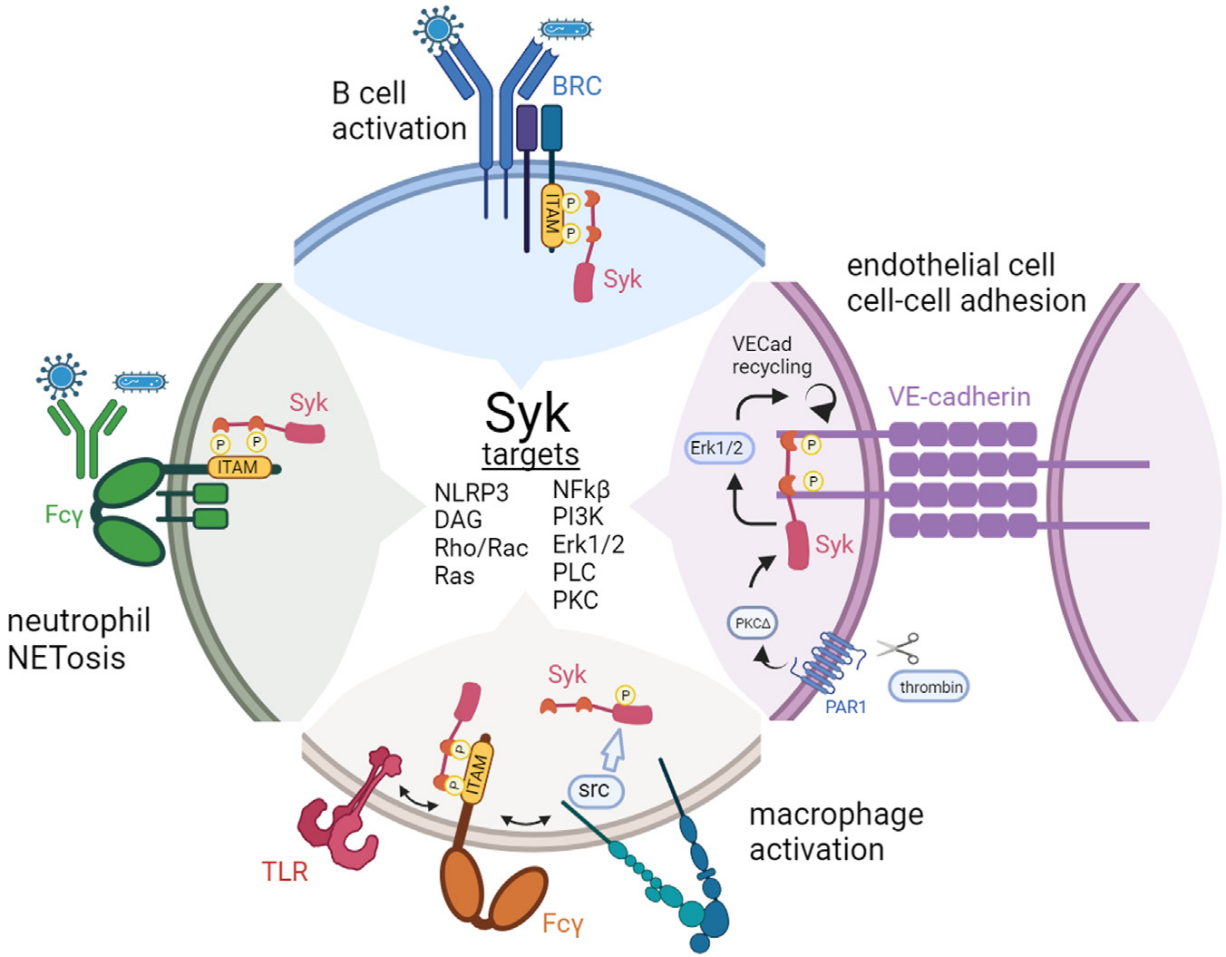
**The many roles of Syk. Syk (*pink*) binds to phosphorylated ITAMs (*yellow*) in B-cell receptors (*blue*) and Fcγ receptors (*green*, *orange*).** Syk can also signal from activated integrins in macrophages (*green/blue*) *via* src, or in conjunction with Toll-like receptors (*pink*). Shadab *et al.* (8) show that Syk, can bind to and regulate VE-cadherin (*purple*). Potential mechanisms include thrombin-induced activation of Syk, which could both target Syk to VE-cadherin and induce Erk1/2-mediated VE-cadherin turnover to decrease endothelial cell-cell adhesion and increase vascular permeability. Created with BioRender.com.

In a recent report in JBC, Shadab *et al.* demonstrated that in addition to its role in the immune cells, Syk has a direct role in endothelial barrier function (8). Using a combination of fostamatinib inhibition and genetic approaches, their study showed that depletion of Syk *in vitro* can partially protect against endothelial barrier dysfunction in response to thrombin or plasma from sepsis patients, supporting the idea that Syk has important endothelial intrinsic functions. Loss of Syk activity decreased VE-cadherin phosphorylation on tyrosine residues 658 and 685 in response to these stimuli, suggesting that Syk performs this phosphorylation directly or is responsible for recruiting Src and/or inhibiting the phosphatase VE-PTP (5). In support of the former interpretation, thrombin induced a complex between Syk and VE-cadherin. Shadab *et al.* also used an innovative technique to induce endothelial-specific knockout of Syk in mice lungs and show that Syk is required for sepsis-induced pulmonary edema and inflammation responses in a sepsis-induced ARDS model *in vivo*. Finally, Syk inhibition by fostamatinib administration was shown to be a viable therapy to control sepsis-induced lung edema, with improved survival in sepsis with fostamatinib. These discoveries are exciting as they suggest repurposing of a currently FDA-approved drug for the treatment of a deadly sepsis-induced lung injury.

Rather than being a burden of poor cellular specificity, treatments that can target both leukocytes and endothelial cells to block vascular inflammation and dysfunction may be a better strategy than those that target only one specific cell type. In this way, a single therapy can address the misregulated cross-talk between the vasculature and immune systems. However, with these advances new mechanistic and clinical questions arise. On the mechanistic side, how is Syk regulating VE-cadherin-mediated barrier function? For example, Shadab *et al.* convincingly show a complex between Syk and VE-cadherin. However, human VE-cadherin does not contain any *bona fide* ITAMs, but does have partial ITAMs (YxxL/I) at 725 and 757. Currently, we do not know whether these cryptic sites can recruit Syk, perhaps as a receptor dimer (Fig. 1), or if another adapter participates. The signaling may also be more complex, as thrombin can induce Erk1/2 activation, which leads to calpain-dependent cleavage and recycling of VE-cadherin (9). Shadab *et al.* also found that Syk depletion lowered Erk1/2 activation by thrombin, suggesting that this pathway may be involved (8). On the clinical side, the findings suggest that some of the promising effects of fostamatinib treatment in other diseases such as COVID-19, autoimmune hemolytic anemia, and hematological malignancies may be in part due to vascular effects (6). Would combinations of Syk inhibitors with other drugs, such as Abl kinase inhibitors, be of value in treating COVID-related ARDS, where results have been mixed (10)? Answering these critical questions awaits further study in the quest for effective ARDS treatments.

## Conflict interest

The author declares that they have no conflicts of interest with the contents of this article.
